# Novel ESCRT functions at kinetochores

**DOI:** 10.18632/aging.101399

**Published:** 2018-03-20

**Authors:** Eleni Petsalaki, George Zachos

**Affiliations:** 1 Department of Biology, University of Crete, Vassilika Vouton, Heraklion 70013, Greece

**Keywords:** ESCRT, Chmp4c, kinetochore, spindle checkpoint, microtubules, RZZ, mitosis

Formation of stable kinetochore-microtubule attachments is essential for accurate chromosome segregation in human cells. This process is monitored by the spindle assembly checkpoint (SAC) that delays sister chromatid separation in the presence of unattached or improperly-attached kinetochores [1]. Kinetochore localization of the Mad1-Mad2 heterotetramer is a major determinant of the spindle checkpoint activity [1]. In metazoans, Mad1-Mad2 recruitment to kinetochores requires the activity of the Rod–ZW10–Zwilch (RZZ) protein complex however, how RZZ is targeted to kinetochores is a matter of active investigation [2,3].

The endosomal sorting complex required for transport (ESCRT) machinery promotes budding and severing of narrow cytosol-filled membrane invaginations from their inner face [4]. Functions of the ESCRT machinery include the biogenesis of multivesicular bodies in endolysosomal sorting, viral budding, plasma membrane wound repair, extraction of defective nuclear pore complexes, neuron pruning, micro- and macroautophagy and nuclear envelop reformation [4]. Furthermore, charged multivesicular body protein 4C (Chmp4c), a human orthologue of the yeast protein Snf7 and an ESCRT-III component, is required for the abscission checkpoint that delays the final cut of the narrow cytoplasmic canal between daughter cells in the presence of trapped chromatin [4]. Importantly, all the above functions involve the conserved membrane-severing activities of the ESCRTs.

**Figure 1:**
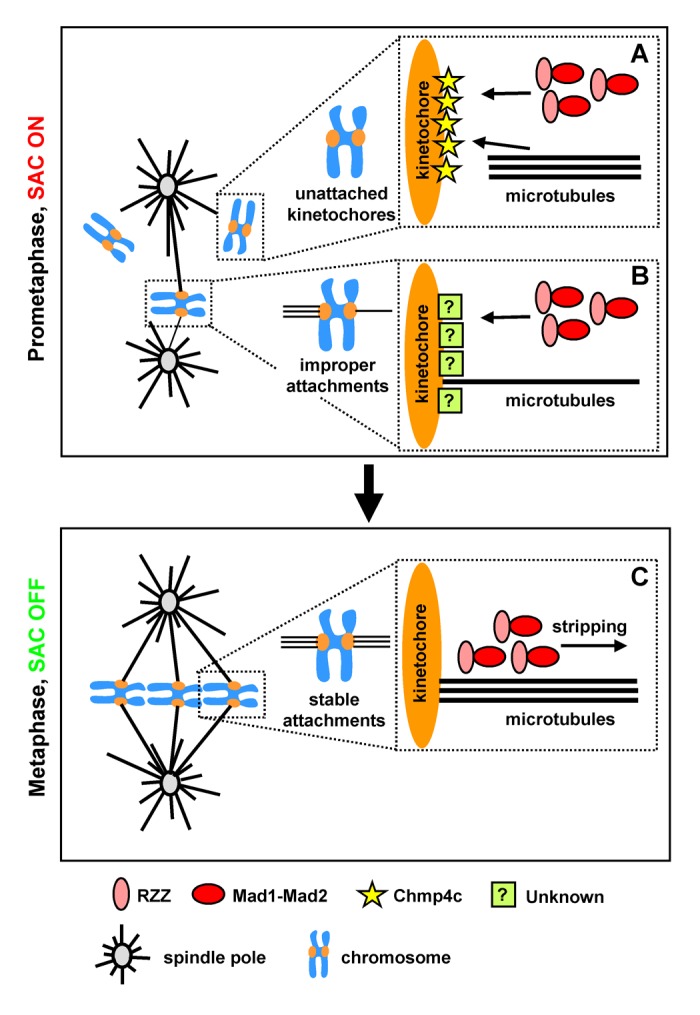
**Newly-identified functions of the ESCRT protein Chmp4c at kinetochores.** In prometaphase, Chmp4c promotes localisation of the RZZ and Mad1-Mad2 complexes to unattached kinetochores (arrow, A) leading to spindle assembly checkpoint (SAC) activation. Chmp4c also promotes stable kinetochore-microtubule interactions (A). In the presence of improper kinetochore-microtubule attachments (B), additional proteins (indicated by a question mark) mediate RZZ-kinetochore targeting. In metaphase (C), Chmp4c is removed from kinetochores leading to RZZ and Mad1-Mad2 stripping and SAC silencing.

In a recent study, we identified novel Chmp4c functions at kinetochores that are independent from its membrane-directed activities [5]. Chmp4c localizes to kinetochores in prometaphase in the absence of spindle poisons or after treatment of cells with a concentration of nocodazole that completely depolymerises spindle microtubules. However, Chmp4c is reduced from kinetochores in chromosomes aligned at the metaphase plate or in prometaphase cells treated with taxol, a spindle drug that stabilizes microtubules and generates improperly attached kinetochore-microtubules. Chmp4c is required for optimal mitotic progression, chromosome alignment and segregation in the absence of spindle poisons, and for mitotic delay when kinetochores are unattached by nocodazole-treatment. Chmp4c binds to ZW10 in cell extracts and *in vitro* and promotes localization of RZZ and Mad1-Mad2 complexes to prometaphase kinetochores. Furthermore, constitutive Chmp4c kinetochore targeting induces an RZZ-dependent checkpoint metaphase arrest, suggesting that Chmp4c kinetochore localization dictates spindle checkpoint activity. These results show that Chmp4c acts as a loading factor for the RZZ to unattached kinetochores (Figure 1A). However, Chmp4c is dispensable for RZZ kinetochore localization and mitotic delay when spindle function is disrupted by taxol, suggesting that additional proteins mediate localisation of RZZ to improperly-attached kinetochores (Figure 1B) [2,3]. Such alternative mechanisms may allow the spindle checkpoint to respond to more than one type of defect ensuring a higher level of chromosome segregation fidelity.

Chmp4c also promotes cold-stable kinetochore-microtubules and Chmp4c-deficient cells exhibit reduced average centromere distance compared with controls [5]. These results show that Chmp4c is required for stable kinetochore-microtubule attachment and optimal pulling forces from kinetochore-microtubules (Figure 1A). Chmp4c may directly bind to kinetochore–microtubules or regulate the KMN protein network that mediates kinetochore–microtubule interactions in human cells [6]. Collectively, these results suggest that Chmp4c coordinates kinetochore-microtubule attachment with spindle checkpoint signalling, perhaps by relaying information about kinetochore-microtubule attachment from microtubule binding to unbound sites [7]. When stable kinetochore-microtubule attachments are formed in metaphase, Chmp4c dissociates from kinetochores leading to dynein-dependent stripping of RZZ and Mad1-Mad2, and SAC silencing (Figure 1C).

The work by Petsalaki et al. [5] clearly establishes a role for Chmp4c in the SAC however, it also raises several important questions. For example, what is the loading factor for the RZZ complex to improperly-attached kinetochores? What is the nature of these “improper” kinetochore-microtubule attachments? How does Chmp4c localise to unattached kinetochores? Are additional ESCRT proteins involved? And how does Chmp4c promote stable kinetochore-microtubule interactions? Investigating these questions will help us further understand the mechanisms of chromosome segregation in human cells.
